# Endothelial microparticles act as novel diagnostic and therapeutic biomarkers of circulatory hypoxia‐related diseases: a literature review

**DOI:** 10.1111/jcmm.13125

**Published:** 2017-03-18

**Authors:** Fan Deng, Shuang Wang, Liangqing Zhang

**Affiliations:** ^1^ Department of Anesthesiology Affiliated Hospital of Guangdong Medical University Zhanjiang Guangdong China; ^2^ Guangdong Medical University Zhanjiang Guangdong China

**Keywords:** endothelial microparticles, hypoxia, circulatory hypoxia, ischaemic, biomarkers

## Abstract

Circulatory hypoxia‐related diseases (CHRDs), including acute coronary syndromes, stroke and organ transplantation, attract increased attention due to high morbidity and mortality. Mounting evidence shows that hypoxia‐induced oxidative stress, coagulation, inflammation and angiogenesis play extremely important roles in the physiological and pathological processes of CHRD‐related vascular endothelial injury. Interestingly, hypoxia, even hypoxia‐induced oxidative stress, coagulation and inflammation can all induce release of endothelial microparticles (EMPs). EMPs, shed from activated or apoptotic endothelial cells (ECs), reflect the degree of EC damage, and elevated EMP levels are found in several CHRDs. Furthermore, EMPs, which play an important role in cell‐to‐cell communication and function, have confirmed pro‐coagulant, proinflammatory, angiogenic and other functions, affecting pathological processes. These findings suggest that EMPs and CHRDs have a very close relationship, and EMPs may help to identify CHRD phenotypes and stratify the severity of disease, to improve risk stratification for developing CHRDs, to better define prophylactic strategies and to ameliorate prognostic characterization of patients with CHRDs. This review summarizes the known and potential roles of EMPs in the diagnosis, staging, treatment and clinical prognosis of CHRDs.

## Introduction

Hypoxia is the lack of oxygen supply to the tissues, resulting in abnormal cell metabolism and function, as well as morphological alterations in the pathological process 1. The aetiology of hypoxia‐related diseases (HRDs) is complex, and many causes can lead to a vicious cycle with further exacerbation and worsening of the disease. Arterial oxygen pressure drop causes hypoxic hypoxia, tissue blood flow reduction results in circulatory hypoxia, haemoglobin reduction leads to hemic hypoxia, and altered bio‐oxidation of tissues causes dysoxidative hypoxia. These are the four major clinical HRD types. Gradually increasing clinical data indicate that incidence and mortality of CHRDs, including myocardial infarction (MI), heart failure (HF), stroke and organ transplantation, are rising with living standard improvement 2. Oxidative stress, inflammation and coagulation are always the causes of disease exacerbation and worsening and constitute important targets for CHRDs treatment.

Interestingly, hypoxia, oxidative stress, inflammation, coagulation can cause different degrees of vascular endothelial injury and stimulate the release of endothelial microparticles (EMPs). The above conditions can induce the release of EMPs both *in vivo* and *in vitro*. EMP levels are elevated in several CHRDs 3, including acute coronary syndromes (ACS) 4, MI and stroke 5. Furthermore, EMPs, as active players in cell‐to‐cell communication, have pro‐coagulant, proinflammatory and angiogenic properties. This implies that EMPs can help to identify CHRD phenotypes and stratify the severity of disease, to improve risk stratification for developing CHRDs, to better define prophylactic strategies and to ameliorate prognostic characterization of patients with CHRDs. Furthermore, as a pathogenic role for EMPs is clearly emerging, the precise description of the mechanisms underlying their formation is likely to prove valuable in identifying much needed novel therapeutic targets.

## Membrane‐derived vesicles

Cell‐derived vesicles can be defined as spherical particles enclosed by a phospholipid bilayer 6. The dimensions of these structures vary and represent one of the characteristics that distinguish the three types of vesicles previously mentioned: exosomes typically range in size between 30 and 100 nm and are released upon fusion of the limiting membrane of multivesicular bodies with the cell surface, microparticles range between 100 and 1000 nm and are formed by the outward blebbing of the plasma membrane with subsequent release after proteolytic cleavage of the cytoskeleton, while apoptotic bodies are between 1 and 3 μm and are formed by indiscriminate plasma membrane blebbing and possess nuclear fragments and histones 7, 8, 9. In addition, the way these vesicles are obtained and what they carry or contain between these vesicles are different 7.

## EMPs

EMPs are 100‐ to 1000‐nm anucleated vesicles formed following cytoskeletal and membrane reorganization, and released from apoptosis or activation of ECs into the extracellular milieu 10, 11. Ischaemia, hypoxia, hyperglycaemia, bacterial lipopolysaccharides, thrombin, C‐reactive protein, active oxygen cluster and urea were shown to induce the release of EMPs from ECs 12. Previous studies found elevated EMP levels in several disease conditions associated with tissue ischaemia or hypoxia 3 such as ACS 13, 14 and stroke 5. Previous findings suggested that acute hypoxia leading to endothelial stress 15 and subsequently apoptosis constitutes a danger signal in case of an acute ischaemic or hypoxic event and can also trigger tissue repair mechanisms 16. Furthermore, certain circulating EMP phenotypes are closely associated with diagnosis, stratification, treatment and prognosis of CHRDs.

### EMP phenotypes

EMPs can be defined according to membrane glycoproteins, as they possess antigens constitutively expressed on ECs such as CD31. The characteristic surface markers of EMPs that indicate ECs origin are numerous and different, with most of them not being specific markers for ECs. Strategies that use a combination of marker protein derived from different cell types have been proposed to resolve these difficulties. Epitopes derived from different cell types used are the positive EC markers (*e.g*. CD31 and CD144) in combination with the absence of the platelet markers CD41 or CD42b 17.

As shown in Table 1 18, the levels of CD31^+^ EMPs, CD105^+^ EMPs and CD144^+^ EMPs appear to increase in apoptotic ECs. In contrast, when the endothelium becomes functionally activated, CD62E^+^, CD54^+^ and CD106^+^ EMP levels are increased. The relative proportion of CD62E^+^/CD31^+^ EMPs rather than absolute levels, distinguishes EMPs released by activated ECs from those derived from apoptotic ECs. More than 10% CD62E^+^/CD31^+^ EMPs indicates that most originate from activated ECs, whereas 1% or less reflects an apoptotic origin 19.

**Table 1:** Endothelial markers used for detecting EMPs

CD marker	Antigen	Expression	Localization on ECs	Expression on ECs	[Refs]
CD31	PECAM‐1	Apoptosis	Intercellular junction (outside of adherence junctions)	Constitutively expressed	19, 88, 89
CD51	Integrin‐αv	Apoptosis	Surface	Constitutively expressed	90
CD54	ICAM‐1	Activation	Surface		89
CD62E	E‐selectin	Activation	Surface	Low on rested ECs; rapidly up‐regulated in response to inflammation	19, 89
CD105	Endoglin	Apoptosis	Surface	Low on resting ECs; up‐regulated once angiogenesis begins	19, 89
CD106	VCAM‐1	Activation			19, 89
CD144	VE‐cadherin	Apoptosis	Adherence junction	Constitutively expressed	19
CD146	MelCAM	Apoptosis	Intercellular junction (outside of adherence junctions) and surface	Constitutively expressed	54

ECs, endothelial cells; EMPs, endothelial microparticles; PECAM, platelet endothelial cell adhesion molecule; MCAM, melanoma cell adhesion molecule; VE, vascular endothelial.

Studies have confirmed that different stimuli can induce the release of different EMPs both *in vitro* and *in vivo*. Although the clinical significance of these differences in EMP release remains not entirely clear, the pattern of EMP release in response to a specific stimulus differs depending on the type of ECs 20. As shown in Table 2, different EMP subtypes may play a role in CHRDs.

**Table 2:** The different EMPs related to CHRDs

EMPs	Diseases	Aspects related to diseases	[Refs]
CD31^+^	CAD	Coronary endothelial function, cardiovascular events, risk stratification, LV dysfunction and endothelium‐dependent vasodilation	13, 64, 65, 66
	ACS	Acute endothelial injury, risk stratification	54, 69
	MI	Recurrence, the size of MaR in STEMI patients, thrombus occlusion	13, 14, 71
	Cerebrovascular atherosclerosis	The EMP level significantly discriminates extracranial and intracranial arterial stenosis	78
CD51^+^	CAD	Assessing EC injury	13
CD54^+^	MI	Treatment with omega‐3	72
	Acute stroke	Severity, lesion volume and outcome of acute ischaemic stroke	5
CD62E^+^	MI	Thrombus occlusion	71
	Aortic stenosis	Systemic inflammatory activity, activated monocytes	91
	Acute stroke	Stroke severity, infarct volume, the risk for cardiovascular morbidities, recurrent events	78, 80
	Cerebrovascular atherosclerosis	The EMP level significantly discriminates extracranial and intracranial arterial stenosis	78
	Heart transplant	As an independent marker of acute allograft rejection	84
CD105^+^	MI	Angiographic lesions, coronary endothelial damage, restoration of the epicardial blood flow	70
	Acute stroke	Severity, lesion volume and outcome of acute ischaemic stroke	5
	SAH	Cerebral vasospasm	79
CD144^+^	CAD	Intermediate lesion	67
	CHF	An independent predictor of future cardiovascular events	74
	Coronary artery stenosis	Cardiac ischaemia	92
	Acute stroke	Severity, lesion volume and outcome	5
	Heart transplant	An independent predictor of cardiac allograft vasculopathy	85
	Liver transplant	Clinical outcome	83
CD146^+^	ACS	Acute endothelial injury	54
	MI	Ischaemic time	71

CAD, coronary artery disease; LV, left ventricular; ACS, acute coronary syndrome; MI, myocardial infarction; STEMI, ST‐segment elevation myocardial infarction; MaR, myocardium at risk; ECs, endothelial cells; SAH, spontaneous subarachnoid haemorrhage; CHF, congestive heart failure.

#### CD31^+^ EMPs

PECAM‐1 EMPs, defined as CD31^+^ EMPs, are concentrated at endothelial junctions. However, the PECAM‐1 marker is also expressed on the surface of platelets, neutrophils and lymphocyte subsets; therefore, platelet‐specific markers, such as CD41 or CD42b, are used to distinguish CD31^+^ EMPs from platelet‐derived MPs. CD31^+^ EMPs are defined as CD31^+^/CD41^−^ or CD42b^−^ EMPs 21, 22. CD31^+^ EMPs may play diverse roles in vascular biology, regulating the platelet function, angiogenesis, T cell and B cell activation, EC permeability and transmigration across the endothelium 23, 24, 25, 26, 27, 28. Therefore, the released EMPs likely reflect the apoptosis of injured ECs.

#### CD51^+^ EMPs

Integrin‐alpha V^+^ EMPs are defined as CD51^+^ EMPs. CD51 forms a heterodimer with an integrin‐β3 chain, such as glycoprotein IIIa (GPIIIa) or CD61, and binds to vitronectin, von Willebrand factor (vWF) and fibronectin 29. CD51 is found on ECs, B lymphocytes, monocytes, macrophages and platelets 30. CD51^+^ EMPs may play important roles in leucocyte homing and rolling and angiogenesis 28, 31.

#### CD54^+^ EMPs

ICAM‐1 EMPs are CD54^+^ EMPs. ICAM‐1 is the cognate ligand of lymphocyte function‐associated antigen‐1 (LFA‐1), and a transmembrane protein found on the surface of inflamed ECs, epithelial cells, monocytes and macrophages, as well as other immune cells including T cells, B cells and antigen‐presenting cells (APCs) 32, 33, 34, 35. The LFA‐1/ICAM‐1 interaction is critical to the firm adhesion of T cells to the vascular endothelium of inflamed tissues and influences the diapedesis and migration of these adhered lymphocytes out of the vasculature directly into adjacent tissues on the ocular surface 36.

#### CD62E^+^ EMPs

E‐selectin is expressed only on ECs, and E‐selectin EMPs are defined as CD62E^+^ EMPs. E‐selectin is rapidly induced on activated ECs a few hours after inflammatory stimulation, whereas VE‐cadherin, PECAM‐1 and MCAM are constitutively expressed on ECs. CD62E^+^ EMPs may play an important role in recruiting leucocytes to the site of injury during inflammation 23, 24, 25, 26, 27, 28. In addition, tumour necrosis factor‐α (TNF‐α) was shown to induce the release of CD62E^+^ EMPs *in vitro* 20, 37. Thus, CD62E^+^ EMP levels may reflect the degree of ongoing endothelial inflammation.

#### CD105^+^ EMPs

Endoglin EMPs are CD105^+^ EMPs. Endoglin (CD105) is a marker of endothelial and mesenchymal stem cells, and component of TGF‐β, BMP‐9 and BMP‐10‐binding receptor complexes 38. CD105 is increasingly expressed on ECs during tumour angiogenesis and inflammation, but have low expression levels on normal ECs 39. Its expression is significantly increased on blood vessel endothelium in ischaemic tissues 38.

#### CD144^+^ EMPs

VE‐cadherin EMPs are CD144^+^ EMPs. VE‐cadherin is expressed only on ECs and functions as a gatekeeper of endothelial junctions 28, 40. Therefore, CD144 is the most specific marker for EMPs 41. In contrast to PECAM‐1 and MCAM, VE‐cadherin is specifically located at adherence junctions 21. In addition, changes in VE‐cadherin localization are associated with neutrophil migration and increased vascular permeability 42. The release of CD144^+^ EMPs may reflect the structural destruction of the endothelium rather than an inflamed lung.

#### CD146^+^ EMPs

MCAM EMPs are CD146^+^ EMPs. MCAM is an adhesion molecule found on ECs and located outside of adherence junctions. MCAM is not only expressed on ECs, but also found on other cell types 43, especially subsets of T and B lymphocytes as well as natural killer (NK) cells under pathological conditions 44, and pericytes 45. Therefore, CD146^+^ EMPs, another indicator of ECs injury, may be closely related to signalling transduction, endothelial permeability, cell migration, angiogenesis and the immune response 46.

### EMPs as messengers

Although EMPs only represent 5–15% of all MPs, they play a vital role in cell‐to‐cell communication, which impacts biological activity. Not only can they be used as a biomarker to distinguish different stimulating factors, disease status and prognosis, they can actively alter the pathological process and disease development.

#### EMPs and prothrombotic response

A study identified an original pathway of EMP release induced by thrombin, involving activation of the Rho‐kinase ROCK‐II by caspase 2 in the absence of cell death 47. This mechanism of EMP generation depends on the activation of nuclear factor (NF)‐κB, whose signalling pathway controls thrombin‐mediated up‐regulation of the inflammatory mediators ICAM‐1 and IL‐8 48.

Thrombin induces the release of EMPs, which in turn have the ability to mediate thrombin generation in many diseases. PS can bind coagulation factors and promote their activation, which may explain why PS^+^ EMPs have pro‐coagulant properties. In addition, EMPs expressing tissue factor (TF), which is the initiator of the extrinsic coagulation pathway, induce TF‐dependent thrombin formation *in vitro* and *in vivo* 49, 50. Furthermore, TF^+^ EMPs bind to monocytes and platelets, amplifying proinflammatory and pro‐coagulant responses 51. Pro‐coagulant EMPs have also been found in atherosclerotic plaques 52, 53 and in patients with ACS 54. Therefore, EMPs can potentially behave as biological vectors in the dissemination of pro‐coagulant potential.

#### EMPs and proinflammatory response

Experiments in cultured ECs have indicated that EMP release is related with that of proinflammatory factors, indicating that release of EMPs and classical inflammatory pathways may have a close relationship in CHRDs 50. Additionally, the interaction between EMPs and ECs triggers proinflammatory responses by increasing ICAM messenger RNA (mRNA) expression as well as soluble ICAM shedding from target cells 55. EMP can amplify the bidirectional relationship between inflammation and thrombosis by conveying cell information. Hence, EMPs represent both a cause and a consequence of the inflammatory response.

#### EMPs as angiogenic signals

Besides their pro‐coagulant and proinflammatory activities, EMPs are also involved in surface supporting plasmin generation by expressing urokinase‐type plasminogen activator (uPA) and its receptor (uPAR), which could counteract EMP generated thrombin and play a pivotal role in maintaining vascular patency and facilitating cell migration and angiogenesis 56, 57. By conveying plasmin, EMPs activate matrix metalloproteinases, which are involved in extracellular matrix degradation and the release of growth factors that play pivotal roles in tissue remodelling and angiogenesis. Consistently, EMPs derived from ischaemic muscle are known to promote endothelial proliferation and vasculogenesis, and TF‐rich EMPs released by microvascular ECs can overcome the consequences of arterial occlusion and tissue ischaemia by promoting post‐ischaemic neovascularization and tissue reperfusion 58, 59.

However, the involvement of EMPs in the angiogenic response and vascular repair remains controversial. In contrast, EMPs reduce endothelial proliferation as well as the formation of new vessels *in vitro* 60. Similarly, high levels of EMPs isolated from human umbilical vein ECs were shown to abolish angiogenesis, while low EMP amounts promote the formation of capillary‐like structures 61. In addition, angiogenic response promotion may also have deleterious effects in cancer spreading, proliferative diabetic retinopathy 62 or atherosclerotic plaque destabilization by promoting intraplaque neovascularization 63.

## EMPs in CHRDs

In the normal physiological state, oxygen in the air flows from the respiratory tract into the alveoli, diffuses to the blood, binds to haemoglobin and is transported by the blood circulation to the body for use by tissue cells. Obstruction of any of these processes can lead to hypoxia. Circulatory hypoxia, including heart failure, shock, embolism and arterial stenosis, represents a decrease in tissue blood flow resulting in tissue oxygen deficiency. EMPs have been investigated in several human circulatory hypoxia diseases as possible pathogenic factors, prognostic markers and therapeutic targets. The data reported in the following sections are summarized in Table 3.

**Table 3:** The EMPs in circulatory hypoxia diseases

Diseases	Changes of EMPs	Major finding	[Ref.]
CAD	CD31^+^/AV^+^ EMPs ↑ (patients *versus* controls)	Increased CD31^+^/AV^+^ EMP counts positively correlated with impairment of coronary endothelial function	64
	CD31^+^/AV^+^ EMPs ↑ (cardiovascular events in stable CAD patients *versus* controls)	The level of circulating CD31^+^/AV^+^ EMPs is an independent predictor of cardiovascular events in stable CAD patients and may be useful for risk stratification	65
	CD144^+^ EMPs ↑ (patients with intermediate lesions *versus* controls)	The levels of CD144^+^ EMPs are significantly higher in the intermediate lesion group	67
	CD31^+^/AV^+^ EMPs ↑ (stable CAD patients with LV dysfunction *versus* those with normal or preserved LV function)	CD31^+^/AV^+^ EMPs correlated inversely with endothelium‐dependent vasodilation in stable CAD patients	66
	CD31^+^ EMPs ↑ & CD51^+^ EMPs ↑ (patients *versus* controls)	EMP assay appears promising for assessing EC injury in CAD	13
	EMP‐P ↑ (stable CAD patients *versus* controls; stable CAD patients/controls *versus* within 1 hr of AMI patients); EMP‐P ↔ (48 hr after the onset of AMI *versus* stable CAD)	Microparticles shed from activated endothelial cells can interact with platelets and form aggregates *in vitro* and *in vivo*	68
ACS	CD31^+^ EMPs ↑ & CD146^+^ EMPs ↑ (patients *versus* stable coronary/non‐coronary patients)	CD31^+^ EMPs and CD146^+^ EMPs suggest a potentially important role for acute endothelial injury in ACS	54
	CD31^+^ EMPs ↑ & CD51^+^ EMPs ↑ (patients *versus* controls); CD31^+^ EMPs ↑& CD51^+^ EMPs ↔ (ACS patients *versus* SA controls)		13
	CD31^+^/CD42b^−^ EMPs ↑ (ACS patients with high‐risk lesions *versus* low‐risk lesions; lesions with thrombi *versus* those without; mild stenosis *versus* severe/without stenosis; first ACS with high‐risk lesions *versus* those with recurrent ACS)	CD31^+^/CD42b^−^ EMPs may be a useful marker in detecting endothelial injury and risk of ACS as defined by angiography	69
MI	CD31^+^ EMPs ↑ (first MI patients *versus* UA/recurring MI)		13
	CD31^+^ EMPs ↑ (patients *versus* controls)		93
	CD105^+^ EMPs ↑ (the occluded coronary artery of STEMI patients *versus* peripheral blood); CD105^+^ EMPs ↓ (restoration of the epicardial blood flow *versus* controls)	CD105^+^ EMPs are elevated in angiographic lesions and correlated with coronary endothelial damage	70
	CD31^+^/CD42^−^ EMPs ↑ & CD144^+^ EMPs ↔ (patients with LAD infarctions *versus* those with other infarct‐related arteries)	Circulating CD31^+^/CD42^−^ EMPs correlate to the size of MaR in patients with STEMI	14
	AV^+^ EMPs ↑ (STEMI patients *versus* controls; patients in the acute phase and in peripheral blood) CD62E^+^/AV^+^ EMPs ↑ & CD31^+^/AV^+^ ↓ (peripheral circulation of STEMI patients *versus* controls); CD62E^+^/AV^+^ EMPs ↑ & CD146^+^/AV^+^ EMPs ↑ (intracoronary blood in STEMI patients *versus* those in peripheral blood); intracoronary CD146^+^ EMPs ↑(IT ≤3 hr *versus* IT >3 hr)	EMPs in peripheral blood may be sensitive markers of the thrombo‐occlusive vascular process developing in the coronary arteries of STEMI patients	71
	CD54^+^ EMPs ↓ (MI in rats with omega‐3 treatment *versus* controls)		72
CHF	CD62E^+^ EMPs ↑ & CD31^+^ EMPs ↑ (patients *versus* controls/post‐transplant patients); CD62E^+^ EMPs ↔ (patients *versus* post‐transplant patients)	Cardiac transplantation is associated with a different pattern of endothelial cell injury than that seen in heart failure. The phenotypic assessment of EMPs in post‐transplant patients is consistent with increased apoptotic activity	73
	CD144^+^ EMPs ↑ (patients *versus* controls)	High‐CD144^+^ EMP levels are an independent predictor of future cardiovascular events	74
CPR	EMP‐P ↑ & EMP‐M ↑ (immediately after CPR *versus* controls); EMP‐M ↑ (24 hr after CPR *versus* controls)		76
	CD62E^+^ EMPs ↑ (24 hr after CPR *versus* controls); CD62E^+^ EMPs ↔ (immediately after CPR *versus* controls)	An ongoing process of endothelial injury, paralleled by a subsequent endothelial regeneration 24 hrs after resuscitation	75
Severe aortic stenosis	CD62E^+^ EMPs ↑ (patients *versus* controls)	EMPs reflecting the activation of ECs but also conferring systemic inflammatory activity were increased in severe aortic stenosis patients and correlated with the number of activated monocytes	91
Coronary artery stenosis	CD144^+^ EMPs ↓ (immediately after dobutamine stress echocardiography in patients *versus* controls)	Cardiac ischaemia leads to reduced circulating EMP levels under cardiac stress	92
Acute stroke	CD105^+^/CD144^+^ EMPs ↑ & CD105^+^/CD54^+^ EMPs ↑ & CD105^+^/PS^+^ EMPs ↑ (patients *versus* controls)	Certain circulating EMP phenotypes may be associated with severity, lesion volume and outcome of acute ischaemic stroke	5
	CD62E^+^ EMPs ↑ & CD31^+^/CD42b^−^ EMPs ↑ & CD31^+^/AV^+^ EMPs ↑ (patients *versus* controls with risk factors)	The levels of CD62E^+^ EMPs were strongly associated with stroke severity and infarct volume	78
	CD62E^+^ EMPs ↑ (patients *versus* controls)	Elevated CD62E^+^ EMP levels increase the risk for cardiovascular morbidities	80
	CD62E^+^/CD42a^−^/AV^+^ EMPs ↑ & CD31^+^/CD42a^−^/AV^+^ EMPs ↑ (recurrent childhood arterial ischaemic stroke *versus* those with no recurrent/controls/cerebral arteriovenous malformation)	Endothelial injury and cellular activation are different in patients with single and recurrent events	94
	CD62E^+^ EMPs ↔ & CD31^+^ EMPs ↔ (stroke mimic patients *versus* acute ischaemic stroke)	EMPs may not be a good marker for acute ischaemic stroke, given the inability to discriminate between stroke mimics and acute ischaemic stroke	82
	CD146^+^/AV^+^ EMPs ↑ & CD62E^+^/AV^+^ EMPs ↑ & CD146^+^/CD62E^+^/AV^+^ EMPs ↑ (acute ischaemic stroke patients *versus* high cardiovascular risk controls)		81
Cerebrovascular atherosclerosis	CD62E^+^ EMPs ↑ & CD31^+^ EMPs ↑ (patients *versus* controls); CD62E^+^ EMPs ↑ & CD31^+^/CD42b^−^ EMPs ↓ & CD31^+^/AV^+^ EMPs ↓ (patients with extracranial arterial stenosis *versus* patients with intracranial arterial stenosis)	The ratio of CD62E^+^ to CD31^+^/CD42b^−^ or CD31^+^/AV^−^ EMP level significantly discriminated extracranial and intracranial arterial stenosis	78
Cerebral Ischaemia	CD31^+^/CD42b^–^ EMPs ↔ (patients *versus* controls)		77
SAH	CD105^+^ EMPs ↑ & CD62E^+^ EMPs ↑ (patients with cerebral vasospasm *versus* controls)	The elevated CD105^+^ EMPs could be a novel biomarker for cerebral vasospasm in SAH patients	79
DIC	CD105^+^ EMPs ↑ & CD31^+^ EMPs ↑ (Septic shock‐induced DIC *versus* controls)	The EMPs are relevant biomarkers of septic shock‐induced DIC and could be used to evaluate early vascular injury	95
Liver Transplant	CD144^+^ EMPs ↑ (patients *versus* patients with a partial hepatectomy)	The levels of circulating EMPs in liver transplant patients dynamically change after surgery and correlate with the clinical outcome	83
Hepatic I/R	AV^+^ EMPs ↑ (4 hr after reperfusion *versus* controls); AV^−^ EMPs ↑ (between 48 and 72 hrs after reperfusion *versus* controls);	EMPs increase after the injury response during the reparative phase and may be important in angiogenesis that occurs in the regenerating liver	96
Heart transplant	CD62E^+^ EMPs ↑ & CD62E^+^ EMPs/CD31^+^ EMPs ↑ & CD31^+^ EMPs ↔ (patients with rejection *versus* controls)	CD62E^+^ EMPs appeared as an independent marker of acute allograft rejection	84
	CD31^+^ EMPs ↑ (patients *versus* controls)		73
	CD144^+^/CD42a^−^ EMPs ↑(patients *versus* controls; patients with CAV *versus* those without CAV)	CD144^+^/CD42a^−^ EMPs have the high discriminative ability between CAV‐positive and CAV‐negative in heart transplant patients.	85
Renal transplant	CD31^+^ EMPs ↑ (patients with cyclosporine treatment *versus* controls)	Cyclosporine causes endothelial cells to release complement‐activating CD31^+^ EMPs *in vivo*	86
	CD31^+^/CD42b^−^ EMPs ↓ (patients with selective end‐stage kidney disease *versus* controls)		87

AV, annexin V; CAD, coronary artery disease; LV, left ventricular; ACS, acute coronary syndrome; MI, myocardial infarction; AMI, acute myocardial infarction; STEMI, ST‐segment elevation myocardial infarction; SA, stable angina; UA, unstable angina; MaR, myocardium at risk; ECs, endothelial cells; CHF, congestive heart failure; EMP‐P, endothelial microparticle–platelet aggregates; EMP‐M, endothelial microparticle–monocyte aggregates; CPR, cardiopulmonary resuscitation; LAD, left anterior descending artery; SAH, spontaneous subarachnoid haemorrhage; CAV, cardiac allograft vasculopathy; DIC, disseminated intravascular coagulation; ↑, increased; ↔, unchanged; ↓, decreased.

### EMPs and coronary artery disease (CAD)

The vascular endothelium and EMPs play pivotal roles in the pathogenesis and clinical manifestations of CAD. Many groups have demonstrated that EMP levels increase significantly in patients with CAD compared with controls 13, 64. Sinning *et al*. found that CD31^+^/AV^+^ EMPs are significantly higher in patients with major adverse cardiovascular and cerebral events (MACCEs) compared with those without such events 65. Furthermore, Bulut *et al*. demonstrated that CD31^+^/AV^+^ EMP levels are significantly higher in stable CAD (SCAD) patients with left ventricular (LV) dysfunction compared with those with normal or preserved LV function 66. These findings imply that circulating CD31^+^/AV^+^ EMP levels constitute an independent predictor of cardiovascular events in patients with SCAD and may be useful for risk stratification; meanwhile, circulating CD31^+^/AV^+^ EMP levels are inversely correlated with endothelium‐dependent vasodilation in patients with SCAD. In addition, Song *et al*. found that CD144^+^ EMP levels are significantly higher in patients with intermediate lesions compared with controls, which suggests that CD144^+^ EMPs level can reflect the severity of coronary stenosis in patients with CAD 67.

In addition, the levels of EMP–platelet aggregates (EMP‐P) were found to be significantly higher in patients with SCAD than controls and early acute myocardial infarction (AMI) patients. EMP‐P may play a role in thrombus generation through their pro‐coagulant activity 68.

### EMPs and ACS

Rupture or plaque fissuring promotes adhesion of platelets and leucocytes and exposes the tissue to promote thrombus formation and key events in the pathogenesis of acute coronary syndromes (ACS). Mallat *et al*. demonstrated that high levels of pro‐coagulant EMPs (CD31^+^ EMPs and CD146^+^ EMPs) are present in the circulating blood of patients with ACS and may contribute to the generation and perpetuation of intracoronary thrombi. In addition, the presence of CD31^+^ EMPs and CD146^+^ EMPs suggest a potentially important role for acute endothelial injury in ACS 54. Bernal‐Mizrachi *et al*. found that CD31^+^/CD42b^−^ EMP levels are significantly higher in ACS patients with high‐risk lesions than those with low‐risk lesions, in lesions with thrombi compared with those without, in ACS patients with mild stenosis compared with those with severe or without stenosis, and in first ACS with high‐risk lesions than those with recurrent ACS 69. These findings imply that CD31^+^/CD42b^−^ EMPs may constitute a useful marker in detecting endothelial injury and risk of ACS as defined by angiography.

### EMPs and MI

Endothelial activation and dysfunction are prominent features of acute myocardial infarction (MI); mounting evidence shows EMPs play an important role in diagnosis and differential diagnosis of MI. Jung *et al*. found that ST‐segment elevation myocardial infarction (STEMI) patients with left anterior descending artery (LAD) occlusion have higher levels of CD31^+^/CD42^−^ EMP than those with other infarct‐related arteries, while CD144^+^ EMP does not differ between the two groups; meanwhile, CD31^+^/CD42^−^ EMP amounts are correlated to myocardium at risk (MaR) and the area under the curve of troponin T levels, but not to infarct size (IS) and CKMB 14. In patients with first MI, CD31^+^ EMP levels were shown to be higher than in those with UA, and significantly elevated compared with the values obtained for patients with recurring MI 13. Morel *et al*. found that CD105^+^ EMP levels are significantly higher in occluded coronary artery specimens than in peripheral blood samples, with restoration of the epicardial blood flow leading to significantly reduced CD105^+^ EMP levels; these findings suggest that CD105^+^ EMPs are elevated in angiographic lesions and correlated with coronary endothelial damage 70. Interestingly, Suades *et al*. found that AV^+^ EMPs are significantly increased in the systemic circulation of STEMI patients compared with controls, with lower amounts of AV^+^ EMPs in peripheral blood of patients recovering from MI at day 3 than in individuals with acute phase disease. The levels of CD62E^+^/AV^+^ EMPs were shown to be significantly higher, with CD31^+^/AV^+^ EMP amounts significantly lower in peripheral circulation of STEMI patients than controls. In addition, CD62E^+^/AV^+^ EMP levels and CD146^+^/AV^+^ EMP amounts are significantly higher in intracoronary blood than in peripheral blood; meanwhile, CD146^+^/AV^+^ EMP amounts were shown to be significantly higher in patients with ischaemic time (IT) ≤3 hr than in those with IT >3 hr 71. These findings imply that EMPs in peripheral blood may be sensitive markers of the thrombo‐occlusive vascular process developing in the coronary arteries of STEMI patients. Interestingly, omega‐3 bolus before reperfusion significantly decreases I/R injury and CD54^+^ EMP release compared with untreated I/R rats 72. Therefore, CD54^+^ EMPs may constitute a good marker for the treatment of prognosis in MI.

### EMPs and CHF

EMPs are reportedly elevated in patients with congestive heart failure (CHF). Garcia *et al*. found CHF patients have significantly higher levels of CD62E^+^ EMP than controls and post‐transplant patients. CD31^+^ EMP levels in CHF patients are significantly higher than in controls. In addition, CHF patients and controls have significantly higher ratios of CD62E^+^ EMPs to CD31^+^ EMPs than post‐transplant patients; no significant differences were found between CHF patients and controls 73. This implies that EMPs may help in differential diagnosis between CHF and heart transplant. Furthermore, Nozaki *et al*. demonstrated that plasma levels of CD144^+^ EMPs can independently predict future cardiovascular events in patients with HF, constituting a potentially useful biomarker of endothelial dysfunction in HF risk stratification 74.

### EMPs and CPR

Ischaemia and reperfusion after cardiopulmonary resuscitation (CPR) induce endothelial activation and systemic inflammatory response, resulting in post‐resuscitation disease. Fink *et al*. found that CD62E^+^ EMP amounts are slightly and non‐significantly increased immediately after CPR compared with values of patients with CAD or healthy controls until 24 hrs after successful CPR 75. In resuscitated patients, significantly enhanced levels of EMP–monocyte and EMP–platelet conjugates were obtained compared with patients in the cardiologic control group and healthy controls immediately after cardiopulmonary resuscitation (CPR); meanwhile, only the levels of EMP–monocyte conjugates show persisting increase within 24 hrs of CPR 76. The above findings indicated that early endothelial damage and ongoing endothelial dysfunction lead to the release of apoptotic EC‐derived EMPs, and more activating cell‐derived EMPs are increased, which may be related to early endothelial cell regeneration, 24 hrs after CPR.

### EMPs and acute stroke (AS)

Cerebral ischaemia is a major complication after acute brain injury, in which endothelial dysfunction is a key player 77. Detailed profiling of EMPs may help better understand the pathogenesis of stroke and determine the related risks. Cerebrovascular atherosclerosis, ischaemia and vasospasm all can increase EMPs to different degrees 77, 78, 79. Simak *et al*. found that certain circulating EMPs may be associated with severity, lesion volume and outcome in acute ischaemic stroke (AIS). Significantly higher CD105^+^/PS^+^/CD41a^−^ EMP counts were observed in AIS patients compared with controls, with three EMP phenotypes (CD105^+^/CD41a^−^/CD45^−^ EMPs, CD105^+^/PS^+^/CD41a^−^ EMPs and CD105^+^/CD54^+^/CD45^−^ EMPs) correlating significantly with brain lesion volume, and CD105^+^/CD54^+^/CD45^−^ EMPs showing the strongest correlation. Admission counts of CD105^+^/CD144^+^ EMPs and CD105^+^/CD41a^−^/CD45^−^ EMPs were correlated significantly with discharge clinical outcome. However, these changes are different between AIS and moderate to severe stroke; all four EMP phenotypes (CD105^+^/CD41a^−^/CD45^−^ EMPs, CD105^+^/CD144^+^ EMPs, CD105^+^/PS^+^/CD41a^−^ EMPs and CD105^+^/CD54^+^/CD45^−^ EMPs) studied were shown to be elevated in the subgroup of moderate to severe stroke patients compared with controls 5. Jung *et al*. demonstrated that CD62E^+^ EMP levels are strongly associated with stroke severity and infarct volume. Furthermore, CD62E^+^ EMP levels were found to be negatively correlated with time since symptom onset and positively associated with National Institutes of Health Stroke Scale (NIHSS) scores. In addition, EMP profiling is important for the diagnosis of intracranial arterial stenosis (ICAS) and extracranial arterial stenosis (ECAS). The ICAS subgroup was shown to have greater CD31^+^/CD42b^−^ and CD31^+^/AV^+^ EMP levels and a lower CD62E^+^ to CD31^+^ to AV^+^ EMP ratio compared with the other subgroups. In contrast, the ECAS subgroup show greater mean CD62E^+^ EMP levels and CD62E^+^ to CD31^+^/AV^+^ EMP ratio compared with the other subgroups 78. Furthermore, Lee *et al*. found that high level of CD62E^+^ EMPs is associated with cardiovascular events in patients with stroke history, suggesting that systemic endothelial activation increases the risk for cardiovascular morbidities 80. Consistently, Chiva‐Blanch *et al*. found that CD146^+^/AV^+^ EMP and CD62E^+^ EMP levels are significantly higher in AIS patients than high cardiovascular risk controls 81. These findings suggest that different EMPs may have distinct biological significances in the pathogenesis of cerebral atherosclerosis, with different diagnostic values.

However, EMPs may not be a good marker for acute ischaemic stroke, given the inability to discriminate between stroke mimics and acute ischaemic stroke. B. Williams *et al*. found CD62E^+^ EMP and CD31^+^ EMP levels as well as the CD62E^+^ EMPs to CD31^+^ EMPs ratio are similar in patients with AIS and stroke mimic patients 82.

### EMPs and organ transplantation

Mounting evidence shows that the levels of circulating EMPs may constitute a biomarker for evaluating the functional status of transplanted organs in clinical practice. Brodsky *et al*. found that the levels of CD144^+^ EMPs are increased more substantially in patients who underwent liver transplantation compared with those administered partial hepatectomy, while circulating EMP levels in liver transplantation patients dynamically change after surgery and correlate with the clinical outcome 83.

In heart transplantation patients, the levels of CD31^+^ EMPs are significantly higher than in controls 73, and CD62E^+^ EMPs appeared to be an independent predictive factor for acute allograft rejection. Morel *et al*. found that CD62E^+^ EMP levels and the ratio of CD62E^+^ EMPs to CD31^+^ EMPs are significantly higher in heart transplantation patients with rejection than those with no rejection, whereas apparently similar CD31^+^ EMP levels were found 84. Cardiac allograft vasculopathy (CAV) is the most important determinant of cardiac allograft survival, and a major cause of death after heart transplantation. Singh *et al*. found the levels of CD144^+^/CD42a^−^ EMPs are significantly higher in heart transplantation patients compared with controls, and higher in heart transplantation patients with CAV than those without. These findings indicated that CD144^+^/CD42a^−^ EMPs have a high discriminative ability between CAV‐positive and CAV‐negative heart transplant patients 85.

In kidney transplantation patients, circulating EMP levels 1 year after transplantation were shown to be significantly lower than pre‐transplantation values. Interestingly, the levels of EMPs in post‐transplantation patients administered cyclosporine and azathioprine immunosuppressive treatment were shown to be significantly lower than those receiving tacrolimus and mycophenolate immunosuppressive treatment. These findings suggest that renal transplant‐related injuries induced release of EMPs is an important mechanism by which systemic insults trigger intravascular complement activation and complement‐dependent renal diseases 86. In addition, circulating EMP levels in renal transplantation patients with selective end‐stage kidney disease are significantly reduced compared with controls. Circulating EMP amounts change with positive and negative peritubular capillary C4d staining on kidney allograft biopsy, again indicating that circulating EMPs constitute a good biomarker for reflecting EC injury in various diseases 87.

## Conclusions

Although knowledge regarding EMPs in the development of clinical disorders is not completely clear, with potential causal or effect relationships between EMP release and particular disease processed still being unravelled, there is little doubt that EMPs are good biomarkers for identifying CHRD phenotypes and assessing disease severity, improving risk stratification for the development of CHRDs to better define prophylactic strategies and allowing a better prognostic characterization of patients with CHRDs.

## Conflict of interest

The authors declare that they have no conflict of interest.

## References

[1] Huang GX , Pan XY , Jin YD , *et al* The mechanisms and significance of up‐regulation of RhoB expression by hypoxia and glucocorticoid in rat lung and A549 cells. J Cell Mol Med. 2016; 20: 1276–86. 26915688 10.1111/jcmm.12809 PMC4929294

[2] Ivan M , Harris AL , Martelli F , *et al* Hypoxia response and microRNAs: no longer two separate worlds. J Cell Mol Med. 2008; 12: 1426–31. 18624759 10.1111/j.1582-4934.2008.00398.x PMC3918058

[3] Blann A , Shantsila E , Shantsila A . Microparticles and arterial disease. Semin Thromb Hemost. 2009; 35: 488–96. 19739039 10.1055/s-0029-1234144

[4] Huisse MG , Lanoy E , Tcheche DJ , *et al* Prothrombotic markers and early spontaneous recanalization in ST‐segment elevation myocardial infarction. Thromb Haemost. 2007; 98: 420–6. 17721626 PMC2237889

[5] Simak J , Gelderman MP , Yu H , *et al* Circulating endothelial microparticles in acute ischemic stroke: a link to severity, lesion volume and outcome. J Thromb Haemost. 2006; 4: 1296–302. 16706974 10.1111/j.1538-7836.2006.01911.x

[6] van der Pol E , Boing AN , Harrison P , *et al* Classification, functions, and clinical relevance of extracellular vesicles. Pharmacol Rev. 2012; 64: 676–705. 22722893 10.1124/pr.112.005983

[7] Gyorgy B , Szabo TG , Pasztoi M , *et al* Membrane vesicles, current state‐of‐the‐art: emerging role of extracellular vesicles. Cell Mol Life Sci. 2011; 68: 2667–88. 21560073 10.1007/s00018-011-0689-3 PMC3142546

[8] Keller S , Sanderson MP , Stoeck A , *et al* Exosomes: from biogenesis and secretion to biological function. Immunol Lett. 2006; 107: 102–8. 17067686 10.1016/j.imlet.2006.09.005

[9] Burger D , Schock S , Thompson CS , *et al* Microparticles: biomarkers and beyond. Clin Sci. 2013; 124: 423–41. 23249271 10.1042/CS20120309

[10] Burger D , Turner M , Munkonda MN , *et al* Endothelial microparticle‐derived reactive oxygen species: role in endothelial signaling and vascular function. Oxid Med Cell Longev. 2016; 2016: 5047954. doi:10.1155/2016/5047954. 27313830 10.1155/2016/5047954 PMC4893592

[11] Schiro A , Wilkinson FL , Weston R , *et al* Endothelial microparticles as conveyors of information in atherosclerotic disease. Atherosclerosis. 2014; 234: 295–302. 24721189 10.1016/j.atherosclerosis.2014.03.019

[12] Yong PJ , Koh CH , Shim WS . Endothelial microparticles: missing link in endothelial dysfunction? Eur J Prev Cardiol. 2013; 20: 496–512. 22496273 10.1177/2047487312445001

[13] Bernal‐Mizrachi L , Jy W , Jimenez JJ , *et al* High levels of circulating endothelial microparticles in patients with acute coronary syndromes. Am Heart J. 2003; 145: 962–70. 12796750 10.1016/S0002-8703(03)00103-0

[14] Jung C , Sorensson P , Saleh N , *et al* Circulating endothelial and platelet derived microparticles reflect the size of myocardium at risk in patients with ST‐elevation myocardial infarction. Atherosclerosis. 2012; 221: 226–31. 22245039 10.1016/j.atherosclerosis.2011.12.025

[15] Jung C , Quitter F , Lichtenauer M , *et al* Increased arginase levels contribute to impaired perfusion after cardiopulmonary resuscitation. Eur J Clin Invest. 2014; 44: 965–71. 25186018 10.1111/eci.12330

[16] Zhang M , Malik AB , Rehman J . Endothelial progenitor cells and vascular repair. Curr Opin Hematol. 2014; 21: 224–8. 24637956 10.1097/MOH.0000000000000041 PMC4090051

[17] Trzepizur W , Martinez MC , Priou P , *et al* Microparticles and vascular dysfunction in obstructive sleep apnoea. Eur Respir J. 2014; 44: 207–16. 24627532 10.1183/09031936.00197413

[18] Takahashi T , Kubo H . The role of microparticles in chronic obstructive pulmonary disease. Int J Chron Obstruct Pulmon Dis. 2014; 9: 303–14. 24707174 10.2147/COPD.S38931 PMC3971913

[19] Tramontano AF , Lyubarova R , Tsiakos J , *et al* Circulating endothelial microparticles in diabetes mellitus. Mediators Inflamm. 2010; 2010: 250476. doi:10.1155/2010/250476. 20634911 10.1155/2010/250476 PMC2904448

[20] Takahashi T , Kobayashi S , Fujino N , *et al* Differences in the released endothelial microparticle subtypes between human pulmonary microvascular endothelial cells and aortic endothelial cells *in vitro* . Exp Lung Res. 2013; 39: 155–61. 23550836 10.3109/01902148.2013.784932

[21] Bardin N . Identification of CD146 as a component of the endothelial junction involved in the control of cell‐cell cohesion. Blood. 2001; 98: 3677–84. 11739172 10.1182/blood.v98.13.3677

[22] Newman PJ , Berndt MC , Gorski J , *et al* PECAM‐1 (CD31) cloning and relation to adhesion molecules of the immunoglobulin gene superfamily. Science. 1990; 247: 1219–22. 1690453 10.1126/science.1690453

[23] Patil S , Newman DK , Newman PJ . Platelet endothelial cell adhesion molecule‐1 serves as an inhibitory receptor that modulates platelet responses to collagen. Blood. 2001; 97: 1727–32. 11238114 10.1182/blood.v97.6.1727

[24] Cicmil M , Thomas JM , Leduc M , *et al* Platelet endothelial cell adhesion molecule‐1 signaling inhibits the activation of human platelets. Blood. 2002; 99: 137–44. 11756163 10.1182/blood.v99.1.137

[25] Falati S , Patil S , Gross PL , *et al* Platelet PECAM‐1 inhibits thrombus formation *in vivo* . Blood. 2006; 107: 535–41. 16166583 10.1182/blood-2005-04-1512 PMC1895610

[26] Muller WA . Leukocyte–endothelial‐cell interactions in leukocyte transmigration and the inflammatory response. Trends Immunol. 2003; 24: 326–33. 10.1016/s1471-4906(03)00117-0 12810109

[27] Woodfin A , Voisin MB , Nourshargh S . PECAM‐1: a multi‐functional molecule in inflammation and vascular biology. Arterioscler Thromb Vasc Biol. 2007; 27: 2514–23. 17872453 10.1161/ATVBAHA.107.151456

[28] Ley K , Laudanna C , Cybulsky MI , *et al* Getting to the site of inflammation: the leukocyte adhesion cascade updated. Nat Rev Immunol. 2007; 7: 678–89. 17717539 10.1038/nri2156

[29] Sosnoski DM , Emanuel BS , Hawkins AL , *et al* Chromosomal localization of the genes for the vitronectin and fibronectin receptors alpha subunits and for platelet glycoproteins IIb and IIIa. J Clin Invest. 1988; 81: 1993–8. 2454952 10.1172/JCI113548 PMC442653

[30] Kokubo T , Uchida H , Choi ET . Integrin α_v_β_3_ as a target in the prevention of neointimal hyperplasia. J Vasc Surg. 2007; 45: 33–8. 10.1016/j.jvs.2007.02.069 PMC1939972 17544022

[31] Sheppard D . Roles of alphav integrins in vascular biology and pulmonary pathology. Curr Opin Cell Biol. 2004; 16: 552–7. 15363806 10.1016/j.ceb.2004.06.017

[32] Marlin SD , Springer TA . Purified intercellular adhesion molecule‐1 (ICAM‐1) is a ligand for lymphocyte function‐associated antigen 1 (LFA‐1). Cell. 1987; 51: 813–9. 3315233 10.1016/0092-8674(87)90104-8

[33] Jóźwik M , Okungbowa OE , Lipska A , *et al* Surface antigen expression on peripheral blood monocytes in women with gynecologic malignancies. BMC Cancer. 2015; 15: 129. doi:10.1186/s12885‐015‐1136‐x. 25880896 10.1186/s12885-015-1136-x PMC4416309

[34] Kim EC , Moon JH , Kang SW , *et al* TMEM126A, a CD137 ligand binding protein, couples with the TLR4 signal transduction pathway in macrophages. Mol Immunol. 2015; 64: 244–51. 25549946 10.1016/j.molimm.2014.12.001

[35] Xie RF , Hu P , Wang ZC , *et al* Platelet‐derived microparticles induce polymorphonuclear leukocyte‐mediated damage of human pulmonary microvascular endothelial cells. Transfusion. 2015; 55: 1051–7. 25565376 10.1111/trf.12952

[36] Springer TA , Dustin ML , Kishimoto TK , *et al* The lymphocyte function‐associated LFA‐1, CD2, and LFA‐3 molecules: cell adhesion receptors of the immune system. Annu Rev Immunol. 1987; 5: 223–52. 3109455 10.1146/annurev.iy.05.040187.001255

[37] Jimenez JJ , Jy W , Mauro LM , *et al* Endothelial cells release phenotypically and quantitatively distinct microparticles in activation and apoptosis. Thromb Res. 2003; 109: 175–80. 12757771 10.1016/s0049-3848(03)00064-1

[38] Smirnov IV , Griazeva IV , Samoilovich MP , *et al* Production and characterization of monoclonal antibodies against human endoglin. Cell Tissue Biol. 2015; 9: 473–82. 26591062

[39] Chrisostomidis C , Konofaos P , Karypidis D , *et al* The impact of Ets‐1 oncoprotein and human endoglin (CD105) on the recurrence of non‐melanoma skin cancers. Int J Dermatol. 2015; 54: 989–95. 26173753 10.1111/ijd.12891

[40] Shaw SK , Bamba PS , Perkins BN , *et al* real‐time imaging of vascular endothelial‐cadherin during leukocyte transmigration across endothelium. J Immunol. 2001; 167: 2323–30. 11490021 10.4049/jimmunol.167.4.2323

[41] Zhang X , Xiu ZM , Li XW . Preparation and characterization of (HAp/SiO_2_)/Ti Biocomposites. Adv Mater Res. 2011; 217–218: 88–92.

[42] Conway D , Schwartz MA . Lessons from the endothelial junctional mechanosensory complex. F1000 Biol Rep. 2012; 4: 1–6. 22238515 10.3410/B4-1 PMC3251317

[43] Lehmann JM , Riethmuller G , Johnson JP . MUC18, a marker of tumor progression in human melanoma, shows sequence similarity to the neural cell adhesion molecules of the immunoglobulin superfamily. Proc Natl Acad Sci USA. 1989; 86: 9891–5. 2602381 10.1073/pnas.86.24.9891 PMC298608

[44] Elshal MF , Khan SS , Takahashi Y , *et al* CD146 (Mel‐CAM), an adhesion marker of endothelial cells, is a novel marker of lymphocyte subset activation in normal peripheral blood. Blood. 2005; 106: 2923–4. 16204154 10.1182/blood-2005-06-2307

[45] Maier CL , Shepherd BR , Yi T , *et al* Explant outgrowth, propagation and characterization of human pericytes. Microcirculation. 2010; 17: 367–80. 20618694 10.1111/j.1549-8719.2010.00038.x PMC2928225

[46] Wang Z , Yan X . CD146, a multi‐functional molecule beyond adhesion. Cancer Lett. 2013; 330: 150–62. 23266426 10.1016/j.canlet.2012.11.049

[47] Sapet C , Simoncini S , Loriod B , *et al* Thrombin‐induced endothelial microparticle generation: identification of a novel pathway involving ROCK‐II activation by caspase‐2. Blood. 2006; 108: 1868–76. 16720831 10.1182/blood-2006-04-014175

[48] Simoncini S , Njock MS , Robert S , *et al* TRAIL/Apo2L mediates the release of procoagulant endothelial microparticles induced by thrombin *in vitro*: a potential mechanism linking inflammation and coagulation. Circ Res. 2009; 104: 943–51. 19265041 10.1161/CIRCRESAHA.108.183285

[49] Combes V , Simon AC , Grau GE , *et al* *In vitro* generation of endothelial microparticles and possible prothrombotic activity in patients with lupus anticoagulant. J Clin Investig. 1999; 104: 93–102. 10393703 10.1172/JCI4985 PMC408397

[50] Leroyer AS , Anfosso F , Lacroix R , *et al* Endothelial‐derived microparticles: biological conveyors at the crossroad of inflammation, thrombosis and angiogenesis. Thromb Haemost. 2010; 104: 456–63. 20664896 10.1160/TH10-02-0111

[51] Sabatier F , Roux V , Anfosso F , *et al* Interaction of endothelial microparticles with monocytic cells *in vitro* induces tissue factor‐dependent procoagulant activity. Blood. 2002; 99: 3962–70. 12010795 10.1182/blood.v99.11.3962

[52] Leroyer AS , Isobe H , Leseche G , *et al* Cellular origins and thrombogenic activity of microparticles isolated from human atherosclerotic plaques. J Am Coll Cardiol. 2007; 49: 772–7. 17306706 10.1016/j.jacc.2006.10.053

[53] Mallat Z , Hugel B , Ohan J , *et al* Shed membrane microparticles with procoagulant potential in human atherosclerotic plaques: a role for apoptosis in plaque thrombogenicity. Circulation. 1999; 99: 348–53. 9918520 10.1161/01.cir.99.3.348

[54] Mallat Z , Benamer H , Hugel B , *et al* Elevated levels of shed membrane microparticles with procoagulant potential in the peripheral circulating blood of patients with acute coronary syndromes. Circulation. 2000; 101: 841–3. 10694520 10.1161/01.cir.101.8.841

[55] Curtis AM , Wilkinson PF , Gui M , *et al* p38 mitogen‐activated protein kinase targets the production of proinflammatory endothelial microparticles. J Thromb Haemost. 2009; 7: 701–9. 19192109 10.1111/j.1538-7836.2009.03304.x

[56] Lacroix R , Sabatier F , Mialhe A , *et al* Activation of plasminogen into plasmin at the surface of endothelial microparticles: a mechanism that modulates angiogenic properties of endothelial progenitor cells *in vitro* . Blood. 2007; 110: 2432–9. 17606760 10.1182/blood-2007-02-069997 PMC2495018

[57] Lozito TP , Tuan RS . Endothelial cell microparticles act as centers of matrix metalloproteinsase‐2 (MMP‐2) activation and vascular matrix remodeling. J Cell Physiol. 2012; 227: 534–49. 21437907 10.1002/jcp.22744 PMC3205212

[58] Arderiu G , Peña E , Badimon L . Angiogenic microvascular endothelial cells release microparticles rich in tissue factor that promotes postischemic collateral vessel formation. Arterioscler Thromb Vasc Biol. 2015; 35: 348–57. 25425620 10.1161/ATVBAHA.114.303927

[59] Leroyer AS , Ebrahimian TG , Cochain C , *et al* Microparticles from ischemic muscle promotes postnatal vasculogenesis. Circulation. 2009; 119: 2808–17. 19451354 10.1161/CIRCULATIONAHA.108.816710

[60] Mezentsev A , Merks RMH , O'Riordan E , *et al* Endothelial microparticles affect angiogenesis *in vitro*: role of oxidative stress. Am J Physiol Heart Circ Physiol. 2005; 289: H1106–14. 15879485 10.1152/ajpheart.00265.2005

[61] Taraboletti G , D'Ascenzo S , Borsotti P , *et al* Shedding of the matrix metalloproteinases MMP‐2, MMP‐9, and MT1‐MMP as membrane vesicle‐associated components by endothelial cells. Am J Pathol. 2002; 160: 673–80. 11839588 10.1016/S0002-9440(10)64887-0 PMC1850663

[62] Chahed S , Leroyer AS , Benzerroug M , *et al* Increased vitreous shedding of microparticles in proliferative diabetic retinopathy stimulates endothelial proliferation. Diabetes. 2010; 59: 694–701. 20009085 10.2337/db08-1524 PMC2828666

[63] Leroyer AS , Rautou PE , Silvestre JS , *et al* CD40 ligand+ microparticles from human atherosclerotic plaques stimulate endothelial proliferation and angiogenesis a potential mechanism for intraplaque neovascularization. J Am Coll Cardiol. 2008; 52: 1302–11. 18929241 10.1016/j.jacc.2008.07.032

[64] Werner N , Wassmann S , Ahlers P , *et al* Circulating CD31 + /annexin V+ apoptotic microparticles correlate with coronary endothelial function in patients with coronary artery disease. Arterioscler Thromb Vasc Biol. 2006; 26: 112–6. 16239600 10.1161/01.ATV.0000191634.13057.15

[65] Sinning JM , Losch J , Walenta K , *et al* Circulating CD31^+^/Annexin V^+^ microparticles correlate with cardiovascular outcomes. Eur Heart J. 2011; 32: 2034–41. 21186238 10.1093/eurheartj/ehq478

[66] Bulut D , Maier K , Bulut‐Streich N , *et al* Circulating endothelial microparticles correlate inversely with endothelial function in patients with ischemic left ventricular dysfunction. J Cardiac Fail. 2008; 14: 336–40. 10.1016/j.cardfail.2007.11.002 18474347

[67] Song R , Chou YI , Kong J , *et al* Association of endothelial microparticle with NO, eNOS, ET‐1, and fractional flow reserve in patients with coronary intermediate lesions. Biomarkers. 2015; 20: 429–35. 26554436 10.3109/1354750X.2015.1094140

[68] Héloire F , Weill B , Weber S , *et al* Aggregates of endothelial microparticles and platelets circulate in peripheral blood. Variations during stable coronary disease and acute myocardial infarction. Thromb Res. 2003; 110: 173–80. 14512078 10.1016/s0049-3848(03)00297-4

[69] Bernal‐Mizrachi L , Jy W , Fierro C , *et al* Endothelial microparticles correlate with high‐risk angiographic lesions in acute coronary syndromes. Int J Cardiol. 2004; 97: 439–46. 15561331 10.1016/j.ijcard.2003.10.029

[70] Morel O , Pereira B , Averous G , *et al* Increased levels of procoagulant tissue factor‐bearing microparticles within the occluded coronary artery of patients with ST‐segment elevation myocardial infarction: role of endothelial damage and leukocyte activation. Atherosclerosis. 2009; 204: 636–41. 19091315 10.1016/j.atherosclerosis.2008.10.039

[71] Suades R , Padro T , Crespo J , *et al* Circulating microparticle signature in coronary and peripheral blood of ST elevation myocardial infarction patients in relation to pain‐to‐PCI elapsed time. Int J Cardiol. 2016; 202: 378–87. 26432487 10.1016/j.ijcard.2015.09.011

[72] Burban M , Meyer G , Olland A , *et al* An intravenous bolus of EPA: DHA 6: 1 protects against myocardial ischemia‐reperfusion‐induced shock. Shock. 2016; 46: 549–56. 27058043 10.1097/SHK.0000000000000624

[73] Garcia S , Chirinos J , Jimenez J , *et al* Phenotypic assessment of endothelial microparticles in patients with heart failure and after heart transplantation: switch from cell activation to apoptosis. J Heart Lung Transplant. 2005; 24: 2184–9. 16364869 10.1016/j.healun.2005.07.006

[74] Nozaki T , Sugiyama S , Sugamura K , *et al* Prognostic value of endothelial microparticles in patients with heart failure. Eur J Heart Fail. 2010; 12: 1223–8. 20817695 10.1093/eurjhf/hfq145

[75] Fink K , Schwarz M , Feldbrügge L , *et al* Severe endothelial injury and subsequent repair in patients after successful cardiopulmonary resuscitation. Crit Care. 2010; 14: R104. doi:10.1186/cc9050. 20525353 10.1186/cc9050 PMC2911749

[76] Fink K , Feldbrugge L , Schwarz M , *et al* Circulating annexin V positive microparticles in patients after successful cardiopulmonary resuscitation. Crit Care. 2011; 15: R251. 22027379 10.1186/cc10512 PMC3334802

[77] Ierssel SHV , Conraads VM , Craenenbroeck EMV , *et al* Endothelial dysfunction in acute brain injury and the development of cerebral ischemia. J Neurosci Res. 2015; 93: 866–72. 25677574 10.1002/jnr.23566

[78] Jung KH , Chu K , Lee ST , *et al* Circulating endothelial microparticles as a marker of cerebrovascular disease. Ann Neurol. 2009; 66: 191–9. 19743467 10.1002/ana.21681

[79] Lackner P , Dietmann A , Beer R , *et al* Cellular microparticles as a marker for cerebral vasospasm in spontaneous subarachnoid hemorrhage. Stroke. 2010; 41: 2353–7. 20814009 10.1161/STROKEAHA.110.584995

[80] Lee ST , Chu K , Jung KH , *et al* Circulating CD62E+ microparticles and cardiovascular outcomes. PLoS ONE. 2012; 7: e35713. doi:10.1371/journal.pone.0035713. 22563392 10.1371/journal.pone.0035713 PMC3338519

[81] Chiva‐Blanch G , Suades R , Crespo J , *et al* Microparticle shedding from neural progenitor cells and vascular compartment cells is increased in ischemic stroke. PLoS ONE. 2016; 11: e0148176. doi:10.1371/journal.pone.0148176. 26815842 10.1371/journal.pone.0148176 PMC4729528

[82] Williams JB , Jauch EC , Lindsell CJ , *et al* Endothelial microparticle levels are similar in acute ischemic stroke and stroke mimics due to activation and not apoptosis/necrosis. Acad Emerg Med. 2007; 14: 685–90. 17606807 10.1197/j.aem.2007.04.009

[83] Brodsky SV , Facciuto ME , Heydt D , *et al* Dynamics of circulating microparticles in liver transplant patients. J Gastrointestin Liver Dis. 2008; 17: 261–8. 18836617

[84] Morel O , Ohlmann P , Epailly E , *et al* Endothelial cell activation contributes to the release of procoagulant microparticles during acute cardiac allograft rejection. J Heart Lung Transplant. 2008; 27: 38–45. 18187085 10.1016/j.healun.2007.09.031

[85] Singh N , Van Craeyveld E , Tjwa M , *et al* Circulating apoptotic endothelial cells and apoptotic endothelial microparticles independently predict the presence of cardiac allograft vasculopathy. J Am Coll Cardiol. 2012; 60: 324–31. 22813611 10.1016/j.jacc.2012.02.065

[86] Renner B , Klawitter J , Goldberg R , *et al* Cyclosporine induces endothelial cell release of complement‐activating microparticles. J Am Soc Nephrol. 2013; 24: 1849–62. 24092930 10.1681/ASN.2012111064 PMC3810078

[87] Qamri Z , Pelletier R , Foster J , *et al* Early posttransplant changes in circulating endothelial microparticles in patients with kidney transplantation. Transpl Immunol. 2014; 31: 60–4. 25008980 10.1016/j.trim.2014.06.006 PMC4141008

[88] Wang JM , Wang Y , Huang JY , *et al* C‐Reactive protein‐induced endothelial microparticle generation in HUVECs is related to BH4‐dependent NO formation. J Vasc Res. 2007; 44: 241–8. 17351328 10.1159/000100558

[89] Ierssel SHV , Craenenbroeck EMV , Conraads VM , *et al* Flow cytometric detection of endothelial microparticles (EMP): effects of centrifugation and storage alter with the phenotype studied. Thromb Res. 2010; 125: 332–9. 20117824 10.1016/j.thromres.2009.12.019

[90] Tramontano AF , O'Leary J , Black AD , *et al* Statin decreases endothelial microparticle release from human coronary artery endothelial cells: implication for the Rho‐kinase pathway. Biochem Biophys Res Commun. 2004; 320: 34–8. 15207698 10.1016/j.bbrc.2004.05.127

[91] Diehl P , Nagy F , Sossong V , *et al* Increased levels of circulating microparticles in patients with severe aortic valve stenosis. Thromb Haemost. 2008; 99: 711–9. 18392329 10.1160/TH07-05-0334

[92] Sinning JM , Jansen F , Hammerstingl C , *et al* Circulating microparticles decrease after cardiac stress in patients with significant coronary artery stenosis. Clin Investigat. 2016; 39: 1–7. 10.1002/clc.22566 PMC6490784 27410166

[93] Morel O , Hugel B , Jesel L , *et al* Circulating procoagulant microparticles and soluble GPV in myocardial infarction treated by primary percutaneous transluminal coronary angioplasty. A possible role for GPIIb‐IIIa antagonists. J Thromb Haemost. 2004; 2: 1118–26. 15219195 10.1111/j.1538-7836.2004.00805.x

[94] Eleftheriou D , Ganesan V , Hong Y , *et al* Endothelial injury in childhood stroke with cerebral arteriopathy. Am Acad Neurol. 2012; 79: 2089–96. 10.1212/WNL.0b013e3182752c7e PMC3511928 23077025

[95] Delabranche X , Boisrame‐Helms J , Asfar P , *et al* Microparticles are new biomarkers of septic shock‐induced disseminated intravascular coagulopathy. Intensive Care Med. 2013; 39: 1695–703. 23793890 10.1007/s00134-013-2993-x

[96] Freeman CM , Quillin RC , Wilson GC , *et al* Characterization of microparticles after hepatic ischemia‐reperfusion injury. PLoS ONE. 2014; 9: e97945. doi:10.1371/journal.pone.0097945. 24879335 10.1371/journal.pone.0097945 PMC4039439

